# Azithromycin and risk of COPD exacerbations in patients with and without *Helicobacter pylori*

**DOI:** 10.1186/s12931-017-0594-x

**Published:** 2017-05-30

**Authors:** Seung Won Ra, Marc A. Sze, Eun Chong Lee, Sheena Tam, Yeni Oh, Nick Fishbane, Gerard J. Criner, Prescott G. Woodruff, Stephen C. Lazarus, Richard Albert, John E. Connett, Meilan K. Han, Fernando J. Martinez, Shawn D. Aaron, Robert M. Reed, S. F. Paul Man, Don D. Sin, on behalf of the Canadian Respiratory Research Network

**Affiliations:** 1 Centre for Heart Lung Innovation, St. Paul’s Hospital, & Department of Medicine (Respiratory Division), University of British Columbia, Don D Sin, Room 8446-1081 Burrard Street, Vancouver, BC V6Z 1Y6 Canada; 2 Ulsan University Hospital, University of Ulsan College of Medicine, Ulsan, South Korea; 3 Department of Microbiology & Immunology, University of Michigan, Ann Arbor, MI USA; 4 Department of Thoracic Medicine and Surgery, Temple University, Philadelphia, PA USA; 5 Department of Medicine, University of California San Francisco, San Francisco, CA USA; 6 Pulmonary Sciences and Critical Care Medicine, University of Colorado, Denver, CO USA; 7 School of Public Health, University of Minnesota, Minneapolis, MN USA; 8 Department of Internal Medicine, University of Michigan, Ann Arbor, MI USA; 9 Joan and Sanford I. Weill Department of Medicine, Weill Cornell Medical College, Cornell University, New York, NY USA; 10 Department of Medicine, University of Ottawa, Ottawa, ON Canada; 11 Division of Pulmonary and Critical Care Medicine, University of Maryland School of Medicine, Baltimore, MD USA

**Keywords:** *Helicobacter pylori*, COPD, Exacerbation, Azithromycin

## Abstract

**Background:**

*Helicobacter pylori* (HP) infection is associated with reduced lung function and systemic inflammation in chronic obstructive pulmonary disease (COPD) patients. Azithromycin (AZ) is active against HP and reduces the risk of COPD exacerbation. We determined whether HP infection status modifies the effects of AZ in COPD patients.

**Methods:**

Plasma samples from 1018 subjects with COPD who participated in the Macrolide Azithromycin (MACRO) in COPD Study were used to determine the HP infection status at baseline and 12 months of follow-up using a serologic assay. Based on HP infection status and randomization to either AZ or placebo (PL), the subjects were divided into 4 groups: HP+/AZ, HP-/AZ, HP+/PL, and HP-/PL. Time to first exacerbation was compared across the 4 groups using Kaplan-Meier survival analysis and a Cox proportional hazards model. The rates of exacerbation were compared using both the Kruskal-Wallis test and negative binomial analysis. Blood biomarkers at enrolment and at follow-up visits 3, 12, and 13 (1 month after treatment was stopped) months were measured.

**Results:**

One hundred eighty one (17.8%) patients were seropositive to HP. Non-Caucasian participants were nearly three times more likely to be HP seropositive than Caucasian participants (37.4% vs 13.6%; *p* < 0.001). The median time to first exacerbation was significantly different across the four groups (*p* = 0.001) with the longest time in the HP+/AZ group (11.2 months, 95% CI; 8.4–12.5+) followed by the HP-/AZ group (8.0 months, 95% CI; 6.7–9.7). Hazard ratio (HR) for exacerbations was lowest in the HP+/AZ group after adjustment for age, sex, smoking status, ethnicity, history of peptic ulcer, dyspnea, previous hospital admission, GOLD grade of severity, and forced vital capacity (HR, 0.612; 95% CI, 0.442–0.846 vs HR, 0.789; 95% CI, 0.663–0.938 in the HP-/AZ group). Circulating levels of soluble tumor necrosis factor receptor-75 were reduced only in the HP+/AZ group after 3 months of AZ treatment (−0.87 ± 0.31 μg/L; *p* = 0.002); levels returned to baseline after discontinuing AZ.

**Conclusions:**

AZ is effective in preventing COPD exacerbations in patients with HP seropositivity, possibly by modulating TNF pathways related to HP infection.

**Electronic supplementary material:**

The online version of this article (doi:10.1186/s12931-017-0594-x) contains supplementary material, which is available to authorized users.

## Background

Persistent systemic inflammation affects ~15 to 20% of patients with chronic obstructive pulmonary disease (COPD), which in turn is associated with an increased risk of exacerbations and mortality [1]. The etiology for this persistent inflammation in COPD, however, is largely a mystery [2]. There is growing evidence that the gastrointestinal tract is a major modulator and driver of inflammation and that the gut-lung axis may be perturbed in COPD [3, 4]. Previous studies have reported an increased prevalence of *Helicobacter pylori* (HP) infection in patients with COPD [5]. Using serum samples from the Lung Health Study (LHS), we recently showed that ~18% of patients in that cohort had serologic evidence for HP infection, which was associated with systemic inflammation and reduced lung function [6]. HP infection may promote persistent low-grade inflammation by up-regulating antigenic stimulation in mucosal surfaces and by skewing the lymphocyte response towards a T helper (Th) lymphocyte 1 bias [7–9].

Interestingly, azithromycin (AZ), which is being increasingly used to prevent exacerbations in COPD has bactericidal activity against HP both *in vitro* and *in vivo* [10, 11]. In general, the immunomodulatory effects of AZ preferentially attenuate Th1 (rather than Th2) responses [12, 13] and decrease tumor necrosis factor (TNF)-α production by human monocytes [14]. However, whether HP modifies the effectiveness of AZ in patients with COPD is unknown. Using data from the MACRO (MACROlide azithromycin to prevent COPD exacerbations) Study [15], we determined the impact of HP infection status on the beneficial effects of AZ in preventing exacerbations in patients with COPD.

## Methods

Details of the MACRO Study design and results have been published previously [15]. The study cohort consisted of 1142 subjects with COPD who were randomized either to azithromycin (AZ, 250 mg) or a placebo tablet (PL) taken daily for 12 months in addition to usual care. The primary outcome of interest was the time to first exacerbation of COPD, defined as a complex of respiratory symptoms (increased or new onset) consisting of two or more of the following: cough, sputum, wheezing, dyspnea, or chest tightness with a duration of at least three days and requiring treatment with antibiotics or systemic steroids or both in combination [15]. At the time of study entry, all subjects had to have been free of an acute exacerbation of COPD (AECOPD) for at least 4 weeks prior to randomization. Subjects were monitored for AECOPDs at clinic visits, which occurred at 3-month intervals, and by monthly telephone contact between each clinic visit.

For the current study, we used blood samples that were available from 1018 subjects to determine the prevalence of HP seropositivity (HP+ or HP-). Plasma samples, collected at enrollment and at the 12 month visit, were used to measure concentrations of immunoglobulin G (IgG) to HP cytotoxin-associated gene A (CagA) antigen using a commercially available ELISA kit (DRG Diagnostics, GmbH, Marburg, Germany) according to the manufacturer’s protocol. Samples with values greater than 18 DU/mL were regarded as positive for HP infection (HP+). Values lower than 18 DU/mL were considered HP negative (HP-) [6]. Using this cutoff value, seroconverters were defined as patients who were seronegative at enrollment but became seropositive at the 12 month visit. Seroreverters were defined as subjects who were seropositive at enrolment but became seronegative at the 12 month visit. A significant reduction in HP antibody was defined as a ≥50% decrease in IgG antibody levels over 12 months [16, 17]. Fifteen patients failed to come in for follow-up visits and were excluded from the remaining analyses. Thus, 1003 subjects were used to evaluate the time to first exacerbation and rate of exacerbation during the 1 year. These 1003 patients were further divided into 4 groups based on their initial HP status and randomization to AZ or PL treatment (HP+/AZ, HP-/AZ, HP+/PL, HP-/PL). To determine whether AZ and HP status modified common biomarkers of systemic inflammation, we related these parameters to plasma levels of C-reactive protein (CRP) and soluble tumor necrosis factor receptor-75 (sTNFR75), which had been previously measured at baseline and at the 3, 12 and 13 (1 month after treatment discontinuation) month visits in the MACRO participants. The details of these measurements have been previously reported [18]. The MACRO study received approval from each participating institution’s local research ethics board and the present study received approval from the University of British Columbia/Providence Health Care Research Ethics Committee (Approval No. H11-00786).

### Statistical analyses

Exacerbation-free survival was assessed using Kaplan-Meier curves stratified by HP serostatus and AZ use, and the groups were compared by a log-rank test. Bootstrap methods were used to generate the confidence intervals (CIs) for median time to the first exacerbation [19]. Pair-wise comparisons were performed to determine the differences in survival rate between each pair of groups using a generalized Wilcoxon test (Breslow test). A Cox proportional hazards regression was performed to adjust for potential confounders including age, sex, smoking status, ethnicity, history of peptic ulcer, dyspnea, previous hospital admission for COPD during past year, Global Initiative for Chronic Obstructive Lung Disease (GOLD) grade of severity of airflow limitation, and forced vital capacity (FVC) on these relationships using HP-/PL as the reference group. The rates of exacerbation were determined by dividing the number of acute exacerbations by the person-years of follow-up, and were compared using both the Kruskal-Wallis test and negative binomial analysis. We used paired t-tests to compare HP antibody levels at baseline vs. the 12 month follow-up in each of the 4 groups. Plasma concentrations of CRP and sTNFR75 levels were log-transformed and paired t-tests were used to determine the differences in concentrations between samples collected at enrollment and at the 3, 12, and 13 month visits within the 4 groups. All statistical analyses were performed using SPSS version 21.0 for Windows (IBM Corp., Armonk, NY, USA) and R version 3.2.2 (available from https://www.r-project.org).

## Results

### Characteristics of the study subjects based on HP status

Plasma samples from 1018 subjects at enrollment were analyzed to determine the prevalence of HP seropositivity; 181 patients (17.8%) had positive titres. HP seroprevalence was substantially lower among Caucasian than non-Caucasian subjects (13.6% vs 37.4%; *p* < 0.001), even though the history of peptic ulcer was similar between the two groups (13.8% vs 13.7%; *p* = 0.985 by Chi-square test). After excluding 15 patients without follow-up visits, the baseline characteristics of the remaining 1003 patients according to HP status are summarized in Table 1. In the HP seropositive group, the proportion of a peptic ulcer history was higher and FVC (%) was lower as compared to HP seronegative group. The patient characteristics stratified by HP status and treatment (HP+/AZ, HP-/AZ, HP+/PL, or HP-/PL) can be found in Additional file 1: Table S1. There were no significant differences in age, sex, smoking status, pack years of smoking, dyspnea grade, hospital admission for COPD or the use of systemic steroids and/or antibiotics past year, lung function, GOLD grade of severity of airflow limitation, or baseline levels of biomarkers (CRP and sTNFR75) between the 4 groups. The proportion of a peptic ulcer history was higher in HP seropositive groups (HP+/AZ and HP+/PL) than HP seronegative groups (HP-/AZ and HP-/PL).

**Table 1:** Characteristics of *Helicobacter pylori* positive and negative patients

	*H. pylori* positive, *n* = 179	*H. pylori* negative, *n* = 824	*p*-value^*^
Age, years	66.2 ± 8.5	65.3 ± 8.7	0.18
Male sex	118 (65.9)	485 (58.9)	0.08
Current smoker	36 (20.1)	175 (21.3)	0.73
Smoking history - Pack years	58.5 ± 32.5	58.3 ± 31.8	0.94
Ethnicity (Caucasian)	112 (62.6)	716 (86.9)	<0.001
Peptic ulcer history	36 (20.1)	102 (12.4)	0.006
Dyspnea (MRC grade)	1.55 ± 0.89	1.53 ± 0.91	0.78
Hospitalization for COPD past year	80 (44.7)	423 (51.3)	0.11
FEV_1_, Liter	1.1 ± 0.5	1.1 ± 0.5	0.25
FEV_1_, % predicted	38.5 ± 15.1	39.9 ± 15.7	0.27
FVC, Liter	2.5 ± 0.8	2.7 ± 0.9	0.07
FVC, % predicted	67.8 ± 16.4	70.7 ± 18.3	0.049
FEV_1_/FVC %	42.5 ± 12.2	42.6 ± 12.8	0.97
GOLD grade, n (%)			0.65
II	42 (23.5)	216 (26.3)	
III	75 (41.9)	344 (42.0)	
IV	62 (34.6)	260 (31.7)	
Baseline biomarker data			
CRP (mg/L)	4.83 ± 3.89	4.91 ± 3.87	0.80
sTNFR75 (μg/L)	8.79 ± 4.41	8.71 ± 4.87	0.85

Data are presented as means ± standard deviation or absolute number (%)

*MRC* medical research council, *FEV*_*1*_ forced expiratory volume in one second, *FVC* forced vital capacity, *GOLD* Global Initiative for Chronic Obstructive Lung Disease, *CRP* C-reactive protein, *sTNFR75* soluble tumor necrosis factor receptor-75

^*^Unpaired t-test or Chi-square test

The results of FEV_1_, FVC, and FEV_1_/FVC are post-bronchodilator values

### Time to first exacerbation in relation to HP status and azithromycin use

**Fig. 1:**
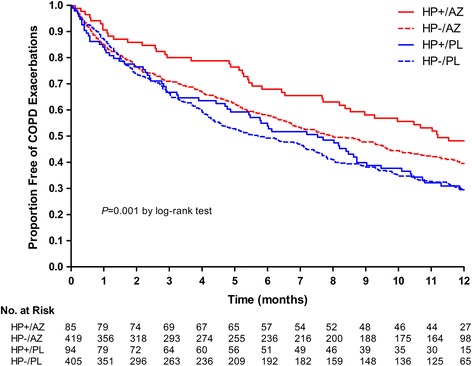
Proportion of participants free from acute exacerbations of chronic obstructive pulmonary disease (COPD). AZ, Azithromycin; PL, Placebo; HP, *Helicobacter pylori*. Pair-wise comparisons between each group showed significant differences in the time to first exacerbation between: The HP+/AZ group versus HP-/AZ group (11.2 months, 95% CI; 8.4–12.5+ vs 8.0 months, 95% CI; 6.7–9.7; *p* = 0.040); The HP+/AZ group versus the HP+/PL group (7.5 months, 95% CI; 4.9–8.8; *p* = 0.006); and The HP+/AZ group versus the HP-/PL group (5.7 months, 95% CI; 4.5–7.2; *p* = 0.001). There was a significant difference between the HP-/AZ and the HP-/PL groups (*p* = 0.020). The remaining pair-wise comparisons were not statistically significant

The median time to first exacerbation was the longest in the HP+/AZ group (11.2 months, 95% CI; 8.4–12.5+), followed by the HP-/AZ group (8.0 months, 95% CI; 6.7–9.7), the HP+/PL group (7.5 months, 95% CI; 4.9–8.8) and the HP-/PL group (5.7 months, 95% CI; 4.5–7.2) with a significant difference across these 4 groups by a log-rank test (*p* = 0.001; Fig. 1). Pair-wise comparisons were performed across the 4 groups using a generalized Wilcoxon test (Breslow test), which showed significant differences in survival function for the time to first exacerbation between the HP+/AZ group and the HP-/AZ group (*p* = 0.04); the HP+/PL group (*p* = 0.006) and the HP-/PL group (*p* = 0.001); and between the HP-/AZ and the HP-/PL groups (*p* = 0.02). A Cox proportional hazards regression was performed to investigate the relationship between the 4 groups in regards to the time to first exacerbation. Hazard ratio (HR) for the time to first exacerbation was the lowest in the HP+/AZ group after adjusting for age, sex, smoking status, ethnicity, history of peptic ulcer, dyspnea, previous hospital admission for COPD during past year, GOLD grade of severity of airflow limitation, and FVC (HR, 0.612; 95% CI, 0.442–0.846; *p* = 0.003 vs HR, 0.789; 95% CI, 0.663–0.938; *p* = 0.007 in the HP-/AZ group vs HR, 1.096; 95% CI, 0.832–1.442; *p* = 0.52 in the HP+/PL group vs HR, 1 in the reference group [HP-/PL]). The rates of exacerbation per patient-year were 1.21, 1.54, 1.73, and 1.85, respectively (*p* = 0.007 by Kruskal-Wallis test; Additional file 1: Table S2). The HP+/AZ group had a 31% lower exacerbation rate compared to the HP-/PL group (*p* = 0.02), whereas the HP-/AZ group had a 14% lower exacerbation compared to the HP-/PL group (*p* = 0.08).

### Change in HP status and antibody levels over 1 year

We also measured HP antibodies in plasma collected at the 12 month visit in 643 subjects. The HP seroprevalence changed from 17.8% (181/1018) to 16.6% (107/643) after 1 year.

#### Seroconversion rates and changes in antibody concentrations

**Fig. 2:**
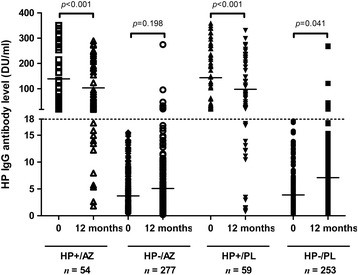
Individual IgG antibody titres to *H. pylori* (HP) CagA antigen in plasma of COPD patients at 0 (baseline) and 12 months. AZ, Azithromycin; PL, Placebo. The horizontal lines denote arithmetic means for individual groups. The differences in antibody concentrations within an individual patient between two time points were compared using a paired t-test. During follow-up, there was a significant decrease in antibody level from baseline (mean ± SEM) in both the HP+/AZ group (−35.42 ± 9.41; *p* < 0.001) and the HP+/PL group (−45.91 ± 11.83; *p* < 0.001) over the year. There was no significant increase in antibody level from baseline in the HP-/AZ group (1.38 ± 1.07; *p* = 0.198). In contrast, there was a significant increase in the HP-/PL group (3.21 ± 1.56; *p* = 0.041)

Of the 530 COPD patients at risk of acquiring HP infection (*i.e*. seronegative at the baseline plasma measurement), 21 (8 from the HP-/AZ group and 13 from the HP-/PL group) became seropositive, resulting in an annual seroconversion rate of 4.0% (Table 2). Overall, there was a significant increase in HP antibody titres over 1 year of follow-up among participants in the HP-/PL group (3.21 ± 1.56 DU/ml; mean ± standard error of mean; *p* = 0.041; Fig. 2). In contrast, the HP antibody titres did not change significantly during follow-up in the HP-/AZ group (1.38 ± 1.07 DU/ml ; *p* = 0.198).

**Table 2:** HP status at 12 months according to baseline HP status and treatment in 643 COPD patients

Baseline HP status and treatment	HP status after 12 months		Total
	Negative *n* = 536	Positive *n* = 107	
HP-/AZ	269 (97.1%)	8 (2.9%)	277 (100%)
HP-/PL	240 (94.9%)	13 (5.1%)	253 (100%)
Total (HP-)	509 (96.0%)	21 (4.0%)	530 (100%)
HP+/AZ	11 (20.4%)	43 (79.6%)	54 (100%)
HP+/PL	16 (27.1%)	43 (72.9%)	59 (100%)
Total (HP+)	27 (23.8%)	86 (76.2%)	113 (100%)

The percentages in the table were calculated row-wise

*HP Helicobacter pylori*, *AZ* azithromycin, *PL* placebo

#### Seroreversion rates and changes in antibody concentrations

The seroreversion rate was not statistically different between AZ and PL use group (20.4% vs 27.1%; *p* = 0.401 by Chi-square test; Table 2). Of the 113 COPD patients who were IgG seropositive at study entry, 27 (11 from the HP+/AZ group, 16 from the HP+/PL group) became seronegative, resulting in an annual seroreversion rate of 23.8% (Table 2); 25 patients (22.1%) demonstrated at least a 50% reduction in their baseline IgG antibody titres at 12 months (Additional file 1: Table S3). In the HP+/AZ group, HP antibody titres decreased significantly during follow-up (−35.42 ± 9.41 DU/ml; *p* < 0.001; Fig. 2). Patients in the HP+/PL group also experienced a significant decline in HP titres during this time (−45.91 ± 11.83 DU/ml; *p* < 0.001).

### Serial change in CRP and sTNFR75 in relation to baseline HP status and treatment

**Fig. 3:**
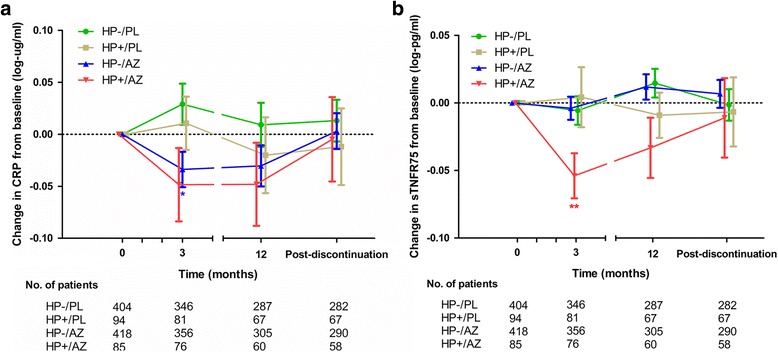
Serial change in blood biomarkers according to baseline *H. pylori* (HP) status and treatment. AZ, Azithromycin; PL, Placebo. The points and error bars indicate the means and standard errors of change in C-reactive protein (CRP) and soluble tumor necrosis factor-75 (sTNFR75) levels from baseline to 3, 12, and 13 months for each of the 4 groups. Paired t-tests were used to determine differences in biomarker concentrations between the three time points and baseline levels. ^*^ *p* < 0.05, ^**^ *p* < 0.01. **a** CRP decreased significantly at 3 months in the HP-/AZ and AZ use group. After stopping AZ at 12 months, CRP returned to baseline levels at 13 months. **b** sTNFR75 levels decreased significantly at 3 months only in the HP+/AZ group and returned to baseline levels after stopping AZ

Plasma CRP and sTNFR75 levels were available for 1001 of the 1003 subjects (99.8%) at baseline and 859 (85.6%), 719 (71.7%), and 697 (69.5%) subjects at 3, 12, and 13 months of follow-up, respectively. We analyzed the mean (*±* SEM) changes in the log-transformed CRP or sTNFR75 levels within subjects from baseline to 3, 12, and 13 months using paired t-tests (Fig. 3). CRP decreased significantly after 3 months in the HP-/AZ group (−0.33 ± 0.18 mg/L; *p* = 0.047) and in the AZ use group (−0.34 ± 0.16 mg/L; *p* = 0.02; Fig. 3 and Additional file 1: Table S4). After discontinuing AZ at 12 months, CRP returned to baseline levels at 13 months in these groups. In terms of sTNFR75, only the HP+/AZ group showed a significant decrease at 3 months (−0.87 ± 0.31 μg/L; *p* = 0.002; Fig. 3, Table 3) and the levels returned to baseline levels after discontinuing AZ. The differences in CRP and sTNFR75 concentrations between the other time points and baseline levels were not significant.

**Table 3:** Effect of azithromycin on changes in sTNFR75 levels at 3 months according to subgroups

Subgroup		sTNFR75 level (μg/L)			*p*-value^*^
	N	Baseline	3 months	Difference	
HP +	76	8.65 ± 0.50	7.78 ± 0.45	−0.87 ± 0.31	0.002
HP -	356	8.52 ± 0.24	8.63 ± 0.32	0.11 ± 0.25	0.64
Total	432	8.54 ± 0.22	8.48 ± 0.27	−0.06 ± 0.21	0.10
Ex-smoker	360	8.73 ± 0.24	8.56 ± 0.31	−0.17 ± 0.24	0.03
Smoker	89	8.10 ± 0.41	8.11 ± 0.43	0.01 ± 0.30	0.89
Age > 65	220	8.52 ± 0.29	8.46 ± 0.42	−0.06 ± 0.36	0.25
Age ≤ 65	229	8.68 ± 0.31	8.48 ± 0.32	−0.20 ± 0.20	0.08
GOLD 2	120	9.07 ± 0.39	9.08 ± 0.67	0.01 ± 0.60	0.15
GOLD 3	183	8.68 ± 0.32	8.49 ± 0.34	−0.19 ± 0.22	0.29
GOLD 4	145	8.12 ± 0.40	7.86 ± 0.40	−0.26 ± 0.27	0.19

Data are presented as means ± standard errors of the mean

*sTNFR75* soluble tumor necrosis factor-75, *GOLD* Global Initiative for Chronic Obstructive Lung Disease, *HP Helicobacter pylori*

^*^Paired t-tests on log-transformed data

## Discussion

The present study is the first to evaluate the effect of azithromycin on COPD exacerbations according to serostatus for *Helicobacter pylori* (HP). The most important findings were that approximately 18% of patients with COPD in the MACRO Study were seropositive for HP and that these individuals experienced the largest reduction in the risk for exacerbation from chronic prophylactic AZ therapy. AZ therapy in these patients was associated with a significant decline in plasma sTNFR75 levels, but not with seroreversion rate as compared to PL use group. Together, these data suggest that AZ therapy alone probably does not eradicate HP infection but may have a significant immunomodulatory role that mitigates the risk of exacerbations in HP seropositive individuals.

It is now well-established and accepted that chronic prophylactic therapy with macrolides such as AZ reduces the risk of exacerbations in patients with COPD. However, many national and international guidelines and strategic documents avoid strong endorsement for their use because of the concerns about long-term side effects, including decreased hearing acuity, arrhythmogenesis, and the possibility of promoting widespread antimicrobial resistance [20, 21]. Given these concerns, it would be highly desirable to develop biomarkers that can identify patients who would benefit the most from these therapies. In a *post hoc* analysis of the MACRO data, Han and colleagues showed that the benefits of AZ were greatest in ex-smokers, older patients, and subjects with mild COPD. However, in their study, reductions in exacerbations were still seen in younger patients and those with higher GOLD grades of airflow limitation, particularly for exacerbations that required treatment with antibiotics and steroids [22]. We extend these findings by demonstrating that the risk of exacerbation is reduced by 39% with AZ in patients who were seropositive to HP (versus only 21% in HP- individuals; *p* = 0.04). Interestingly, using the same cohort, Woodruff et al. found that a decline in sTNFR75 concentrations over 3 months identified COPD patients who benefited from AZ therapy [18]. We extend these findings by demonstrating that the largest (and most significant) reductions in circulating sTNFR75 levels after 3 months were observed in HP+ patients who were treated with AZ (*N* = 76; −0.87 ± 0.31 μg/L; *p* = 0.002), followed by ex-smokers (*N* = 360; −0.17 ± 0.24 μg/L; *p* = 0.03; Table 3). However, AZ therapy was also beneficial in HP seronegative individuals (although the impact was less striking than those who were HP+), suggesting that the mechanisms and pathways by which AZ mitigates exacerbations in COPD are diverse and complicated and extend beyond those with HP seropositivity.

In contrast to sTNFR75, CRP concentration at 3 months was significantly reduced in the AZ group (*N* = 432; −0.34 ± 0.16 mg/L; *p* = 0.02; Fig. 3, Additional file 1: Table S4) and not reduced in the HP+/AZ group (*N* = 76; −0.40 ± 0.38 mg/L; *p* = 0.17), suggesting that potential mechanisms for the reduction in CRP levels include an anti-inflammatory effect independent of the presence of HP infection. Previously it was shown that there was an increase in CRP level in mild to moderate COPD patients who were stable and HP positive [6], whereas the present study showed no significant difference in CRP or sTNFR75 levels between HP negative and positive COPD patients. In addition, there was no significant difference in the time to first exacerbation or rates of exacerbations between the HP+/PL and HP-/PL groups (Fig. 1). We postulate that this may reflect a selection bias towards inclusion of individuals prone to high systemic inflammation and exacerbation in the MACRO Study. It is likely that these individuals had multiple drivers of exacerbations (not just HP infection), which may have obscured the effects of HP infection on systemic inflammation and the overall risk of exacerbations in patients who received placebo during follow-up. Consistent with this notion, the average CRP values in the MACRO Study participants were significantly higher compared to those of previous studies, which had enrolled stable COPD patients [1, 6].

Seropositivity to HP, and annual rates of seroconversion and reversal, is not well-known in patients with COPD. Our previous study using serum samples from the lung health study (LHS) and ELISA plates coated with CagA protein demonstrated a cross-sectional prevalence of HP seropositivity of 17.6% [6]. However, this study was limited in that serum samples were collected in the 1990’s before the widespread availability of triple therapy for HP eradication. Here, using a more contemporaneous cohort (MACRO), we report a similar HP seropositivity rate despite advances in diagnosis and availability of effective therapies for HP. It is also notable that in the MACRO Study we found a substantially higher rate of HP seropositivity among non-Caucasian subjects than Caucasian subjects (37.4% vs 13.6%). Considering that approximately 60% of Western HP strains demonstrate CagA antigen expression, these data are consistent with results from the National Health and Nutrition Examination Survey (1999–2000) conducted in US adults, which used HP antigen coated ELISA plates (Wampole Laboratories, Princeton, New Jersey) and reported a higher age-standardized seroprevalence of HP in Mexican Americans (64.0%) and non-Hispanic blacks (52.0%) compared with Caucasians (21.2%) [23]. Despite the difference of HP seropositivity between Caucasian and non-Caucasian subjects, the proportion with a peptic ulcer history was similar between two groups in our study, suggesting that additional studies would be required to find the mechanism to explain the disparities among ethnicities who may have different dietary cultures and diverse intestinal microbiota communities. AZ may have differential effect on the intestinal microbiota including *helicobacter* genus. The overall annual seroconversion rate in our study subjects was 4% with rates numerically lower in the AZ group compared to the PL group. A 4% annual seroconversion rate in our study subjects is higher than previously reported in high-income countries (~0.01–1% per year in the general adult population) [24–27]. A previous study suggests that older age and smoking are associated with an increased risk of HP [28], possibly due to immune dysregulation and senescence. The overall annual seroreversion rate in our study subjects was 23.8% (27/113) and the rate between the PL and AZ groups was not statistically different. In line with the high rate of seroconversion in our study subjects, seroreversion rate was even higher than other studies performed in the general adult population [24, 27, 29]. Kuipers et al. documented that HP positivity reverted in only 3 of 56 subjects in the absence of specific antimicrobial therapy over 11 years [25]. Since the many of our study subjects were using home oxygen therapy and some had advanced COPD, they experienced frequent exacerbations leading to repeated exposures to antibiotics and steroids during the study period.

Importantly, in the HP-/AZ group, there was no significant increase in HP antibody titres over the 12 month period; by contrast, there was a significant increase in HP antibody titres in the HP-/PL group, suggesting that AZ use prevented significant increases in HP antibody levels in HP- patients. Moreover, although AZ has some *in vitro* and *in vivo* activity against HP, we did not find a significant difference in the rate of seroreversion between the PL and AZ groups. We also used ≥50% decline in titres at 12 months of follow-up compared to baseline levels as indirect evidence for HP eradication [16, 17] since absolute antibody titres remain in the positive range for 1 to 4 years after successful eradication [30, 31]. Interestingly, HP+ patients who experienced at least one exacerbation during the 12 month follow-up were more likely to demonstrate a ≥50% reduction in HP titres during follow-up compared to those who did not experience any exacerbations (30.1% eradication rate vs 7.5%; *p* = 0.006; Additional file 1: Table S3). Again, this raises the possibility that antibiotic exposure during exacerbations may have played a significant role in decreasing antibody levels. Consistent with this hypothesis, a previous study showed that children who had been treated with antibiotics demonstrated lower risk of HP infection compared with those who had never been treated with antibiotics (12.5% vs 30%) [32]. Together, these data suggest that although AZ monotherapy probably does not prevent new HP infection or eradicate existing infection, it likely downregulates the host’s immune responses [10, 11] and dampens macrophage responses and Th1 immunity related to TNF pathways [12–14].

### Limitations of the study

There were several limitations that should be noted in the current study. First, we do not know how many patients with an HP infection developed resistance to macrolides after 1 year of AZ exposure. HP readily becomes resistant to macrolides when given alone, and short-term AZ monotherapy is not considered sufficient for HP eradication [33–35]. Moreover, we had no available data on the presence of gastric diseases or symptoms, or signs that could indicate active HP infection, though we did determine HP serostatus by using the CagA antigen, which is a well-known virulent strain causing more severe gastroduodenal disease [36]. However, to date, AZ monotherapy for COPD patients with HP seropositivity cannot be advocated without further study. For those who are seropositive, HP infection should be further explored and treated with the appropriate triple therapy regimen. Second, we did not have detailed information regarding the use of prescription or over-the-counter antisecretory drugs, which may be responsible for prevention (or even treatment) of COPD exacerbations in patients with gastric symptoms and could have affected HP antibody levels in our patient population. Third, the use of antibiotics or steroids other than AZ could also have affected HP titres in our study subjects. Fourth, the present study used a retrospective design and did not focus on the potential pathogenic mechanisms underlying the association between HP infection and COPD exacerbation. It is well known that HP can stimulate the release of a variety of pro-inflammatory cytokines, including interleukin-1 (IL-1), IL-8 and TNF-α [37, 38] and these cytokines are also thought to be involved in the pathogenesis of COPD [39, 40]. Therefore, HP infection might play a pro-inflammatory role and be a cofactor in the pathogenesis of COPD exacerbation. Finally, although we demonstrated differential effect of AZ therapy on the risk of exacerbation in HP seropositive COPD patients, the mechanism by which this occurs is unknown. We posit that AZ suppresses (but not fully eradicates) HP infection, which in turn, may downregulate the TNF pathway. Experimental studies will be required in the future to tease out the full mechanisms to explain the beneficial effects of AZ on HP infection in COPD patients.

## Conclusions

HP infection status was associated with differential effects of AZ in COPD patients with frequent exacerbations, highlighting the importance of evaluating HP status in these group. Chronic AZ monotherapy suppressed HP antibody levels after 1 year of treatment, but did not significantly modify the rate of seroreversion in the HP+ group. The HP+/AZ group exhibited a decline in sTNFR75 levels at 3 months. Patients who were HP seropositive experienced a significantly greater protection against exacerbation from chronic AZ therapy than those who were HP seronegative. A larger prospective study is needed to definitively prove the benefit of eradication of HP with triple therapy in seropositive HP patients with COPD, who frequently exacerbate.

## Additional file

Additional file 1: Table S1. Patient characteristics according to *Helicobacter pylori* status and treatment. **Table S2.** Effect of *Helicobacter pylori* status at enrollment and treatment on the rates of exacerbation per person-year. **Table S3.** Comparison of the proportion of subjects that showed significant reduction in HP antibody level at 12 months in 113 COPD patients. **Table S4.** Effect of treatment on changes in CRP level from baseline to 3 months according to HP status. (DOCX 27 kb)

## Abbreviations

AECOPD: Acute exacerbation of COPD
AZ: Azithromycin
BD: Bronchodilator
CagA: Cytotoxin-associated gene A
CI: Confidence interval
COPD: Chronic obstructive pulmonary disease
CRP: C-reactive protein
FEV_1_: Forced expiratory volume in one second
FVC: Forced vital capacity
GOLD: Global Initiative for Chronic Obstructive Lung Disease
HP: *Helicobacter pylori*
HR: Hazard ratio
LHS: Lung Health Study
MACRO: MACROlide azithromycin to prevent COPD exacerbations
PL: Placebo
SEM: Standard error of the mean
sTNFR75: Soluble tumor necrosis factor receptor-75
Th: T helper
TNF: Tumor necrosis factor
