# Supplementary material for: Azithromycin and risk of COPD exacerbations in patients with and without Helicobacter pylori
Source: Respir Res. 2017 May 30;18:109. doi: 10.1186/s12931-017-0594-x (PMC5450077; doi:10.1186/s12931-017-0594-x)
Supplement: Additional file 1: Table S1. — Patient characteristics according to Helicobacter pylori status and treatment. Table S2. Effect of Helicobacter pylori status at enrollment and treatment on the rates of exacerbation per person-year. Table S3. Comparison of the proportion of subjects that showed significant reduction in HP antibody level at 12 months in 113 COPD patients. Table S4. Effect of treatment on changes in CRP level from baseline to 3 months according to HP status. (DOCX 27 kb) [file 12931_2017_594_MOESM1_ESM.docx]

**ONLINE SUPPLEMENT**

**Azithromycin and risk of COPD exacerbations in patients**

**with and without *Helicobacter pylori***

**Seung Won Ra^1,2^, Marc A. Sze^3^, Eun Chong Lee^1^, Sheena Tam^1^, Yeni Oh^1^, Nick Fishbane^1^, Gerard J. Criner^4^, Prescott G. Woodruff^5^, Stephen C. Lazarus^5^, Richard Albert^6^, John E. Connett^7^, Meilan K. Han^8^, Fernando J. Martinez^9^, Shawn D. Aaron^10^, Robert M. Reed^11^, SF Paul Man^1^, Don D. Sin^1^ and on behalf of the Canadian Respiratory Research Network**

**^1^**Centre for Heart Lung Innovation, St. Paul’s Hospital, & Department of Medicine (Respiratory Division), University of British Columbia, Vancouver, BC, Canada

**^2^**Ulsan University Hospital, University of Ulsan College of Medicine, Ulsan, South Korea

**^3^**Department of Microbiology & Immunology, University of Michigan, Ann Arbor, MI, USA

**^4^**Department of Thoracic Medicine and Surgery, Temple University, Philadelphia, PA, USA

**^5^**Department of Medicine, University of California San Francisco, San Francisco, CA, USA

**^6^**Pulmonary Sciences and Critical Care Medicine, University of Colorado, Denver, CO, USA

**^7^**School of Public Health, University of Minnesota, Minneapolis, MN, USA

**^8^**Department of Internal Medicine, University of Michigan, Ann Arbor, MI, USA

**^9^**Joan and Sanford I. Weill Department of Medicine, Weill Cornell Medical College, Cornell University, New York, NY, USA

**^10^**Department of Medicine, University of Ottawa, Ottawa, ON, Canada

**^11^**Division of Pulmonary and Critical Care Medicine, University of Maryland School of Medicine, Baltimore, MD, USA

**Address for correspondence:**

Don D Sin, Room 8446 - 1081 Burrard Street, Vancouver, BC, Canada V6Z 1Y6;

Tel: +1-604-806-8395

Fax: +1-604-806-9274

E-mail: [Don.Sin@hli.ubc.ca](mailto:Don.Sin@hli.ubc.ca)

**METHODS**

**Patients and Study design**

Eligibility criteria included a clinical diagnosis of COPD (defined as having a smoking history of at least 10 pack-years, a ratio of postbronchodilator forced expiratory volume in one second [FEV_1_] to forced vital capacity of <70%, and a postbronchodilator FEV_1_ of <80% of the predicted value), and age greater than or equal to 40 years. To enrich the study population for participants who were likely to experience an acute exacerbation of COPD (AECOPD) during follow-up, only those patients who had experienced one or more AECOPD within the previous 12 months or who were using continuous supplemental domiciliary oxygen therapy, were included in the study. Blood was drawn from consenting participants at enrollment (baseline) and at 3, 12, and 13 (1 month after treatment discontinuation) months following randomization.

Blood samples were centrifuged and plasma was transferred to the main coordinating center for storage in −70°C freezers.

**RESULTS**

The patient characteristics stratified by HP status and treatment (HP+/AZ, HP-/AZ, HP+/PL, or HP-/PL) are presented in **Table S1**. The rates of COPD exacerbation according to HP status and azithromycin use are described in **Table S2**. A total of 1,486 exacerbations occurred among the 1,003 participants during the study, 95 among the 85 participants in the HP+/AZ group, 592 among the 419 participants in the HP-/AZ group, 157 among the 94 participants in the HP+/PL group, and 642 among the 405 participants in the HP-/PL group. **Table S3** shows a comparison of the proportion of subjects that showed a significant reduction in HP antibody level at 12 months, in the 113 COPD patients who were HP positive at baseline.

**Table S1** Patient characteristics according to *Helicobacter pylori* status and treatment

|  | **HP+/AZ**  *n* = 85 | **HP-/AZ**  *n* = 419 | **HP+/PL**  *n* = 94 | **HP-/PL**  *n* = 405 | *p*-value^*^ |
| --- | --- | --- | --- | --- | --- |
| Age, years | 65.9 ± 8.4 | 64.7 ± 8.8 | 66.5 ± 8.5 | 65.9 ± 8.5 | 0.12 |
| Male sex | 54 (63.5) | 249 (59.4) | 64 (68.1) | 236 (58.3) | 0.31 |
| Smoking history, pyrs | 60.1 ± 37.3 | 56.9 ± 30.4 | 57.1 ± 27.4 | 59.8 ± 33.2 | 0.55 |
| Current smoker | 17 (20.0) | 90 (21.5) | 19 (20.2) | 85 (21.0) | 0.98 |
| Ethnicity (Caucasian) | 52 (61.2) | 369 (88.1) | 60 (63.8) | 347 (85.7) | < 0.001 |
| Peptic ulcer history | 18 (21.2) | 46 (11.0) | 18 (19.1) | 56 (13.8) | 0.03 |
| Dyspnea (MRC) | 1.51 ± 0.85 | 1.49 ± 0.91 | 1.59 ± 0.92 | 1.56 ± 0.91 | 0.67 |
| Hospitalization for COPD past year | 40 (47.1) | 211 (50.4) | 40 (42.6) | 212 (52.3) | 0.35 |
| Systemic steroids or antibiotics use past year | 65 (76.5) | 355 (84.7) | 78 (83.0) | 350 (86.4) | 0.14 |
| FEV_1_, Liter | 1.0 ± 0.5 | 1.1 ± 0.5 | 1.1 ± 0.5 | 1.1 ± 0.5 | 0.49 |
| FEV_1_, % predicted | 37.7 ± 15.4 | 39.8 ± 15.6 | 39.2 ± 14.8 | 40.0 ± 15.8 | 0.64 |
| FVC, Liter | 2.4 ± 0.8 | 2.7 ± 0.9 | 2.6 ± 0.9 | 2.6 ± 0.9 | 0.053 |
| FVC, % predicted | 66.0 ± 16.8 | 71.3 ± 19.0 | 69.5 ± 15.9 | 70.2 ± 17.5 | 0.10 |
| FEV_1_/FVC % | 42.8 ± 12.9 | 42.4 ± 13.0 | 42.3 ± 11.7 | 42.8 ± 12.7 | 0.98 |
| GOLD grade, n (%) |  |  |  |  | 0.97 |
| Ⅱ | 19 (22.3) | 111 (26.6) | 23 (24.5) | 105 (26.1) |  |
| Ⅲ | 35 (41.2) | 177 (42.3) | 40 (42.5) | 167 (41.6) |  |
| Ⅳ | 31 (36.5) | 130 (31.1) | 31 (33.0) | 130 (32.3) |  |
| CRP (mg/L) | 4.71 ± 3.72 | 4.82 ± 3.64 | 4.94 ± 4.06 | 5.00 ± 4.10 | 0.88 |
| sTNFR75 (µg/L) | 8.42 ± 4.22 | 8.56 ± 4.64 | 9.11 ± 4.57 | 8.88 ± 5.10 | 0.60 |

Data are presented as mean ± standard deviation or absolute number (%).

^*^One way ANOVA or Chi-square test

HP, *Helicobacter pylori*; AZ, azithromycin; PL, placebo; pyrs, pack years; MRC, medical research council; FEV_1_, forced expiratory volume in one second; FVC, forced vital capacity; GOLD, Global Initiative for Chronic Obstructive Lung Disease; CRP, C-reactive protein; sTNFR75, soluble tumor necrosis factor receptor-75.

The results of FEV_1_, FVC, and FEV_1_/FVC are post-bronchodilator values.

**Table S2** Effect of *Helicobacter pylori* status at enrollment and treatment on the rates of exacerbation per person-year

|  | HP+/AZ  *n* = 85 | HP-/AZ  *n* = 419 | HP+/PL  *n* = 94 | HP-/PL  *n* = 405 |
| --- | --- | --- | --- | --- |
| Rates of exacerbations^*^ | 1.21 ± 0.18 | 1.54 ± 0.10 | 1.73 ± 0.20 | 1.85 ± 0.14 |
| Negative binomial analysis^#^ |  |  |  |  |
| Rate ratios (95% CI) | **0.69 (0.50-0.94)** | 0.86 (0.73-1.02) | 1.01 (0.79-1.29) | 1.00 |
| *P*-value | ***p* = 0.02** | *p* = 0.08 | *p* = 0.96 | Reference |

Data are presented as means ± standard errors of the mean or rate ratio (95% CI).

^*^*P*-value = 0.007 by Kruskal-Wallis test.

^a^Adjusted for age, sex, ethnicity, smoking status, and FEV_1_.

HP, *Helicobacter pylori*; AZ, azithromycin; PL, placebo; CI, confidence interval.

**Table S3** Comparison of the proportion of subjects that showed significant reduction in HP antibody level at 12 months in 113 COPD patients

|  | Significant HP antibody reduction at 12 months | | *p*-value^a^ |
| --- | --- | --- | --- |
|  | No  n = 88 | Yes  n = 25 |  |
| Baseline HP status and treatment |  |  | 0.073 |
| HP+/AZ | 46 (85.2%) | 8 (14.8%) |  |
| HP+/PL | 42 (71.2%) | 17 (28.8%) |  |
| Any exacerbation for 12 months |  |  | 0.006 |
| No | 37 (92.5%) | 3 (7.5%) |  |
| Yes | 51 (69.9%) | 22 (30.1%) |  |

^a^Chi-square test.

A difference of 50% or less from the baseline titre can be considered a significant HP antibody decrease. HP, *Helicobacter pylori*; AZ, azithromycin; PL, placebo.

**Table S4** Effect of treatment on changes in CRP level from baseline to 3 months according to HP status

| Subgroup |  | CRP level (mg/L) | | | *p*-value^*^ |
| --- | --- | --- | --- | --- | --- |
|  | N | Baseline | 3 months | Difference |  |
| HP+/AZ | 76 | 4.63 ± 0.43 | 4.23 ± 0.40 | -0.40 ± 0.38 | 0.17 |
| **HP-/AZ** | **356** | **5.04 ± 0.20** | **4.71 ± 0.20** | **-0.33 ± 0.18** | **0.047** |
| **Total (AZ)** | **432** | **5.00 ± 0.18** | **4.62 ± 0.18** | **-0.34 ± 0.16** | **0.02** |
| HP+/PL | 81 | 5.13 ± 0.45 | 5.00 ± 0.39 | -0.14 ± 0.31 | 0.68 |
| HP-/PL | 346 | 4.95 ± 0.22 | 5.37 ± 0.24 | 0.42 ± 0.25 | 0.14 |
| Total (PL) | 427 | 4.98 ± 0.20 | 5.30 ± 0.21 | 0.31 ± 0.21 | 0.13 |

Data are presented as mean ± standard error of the mean.

^*^Paired t-tests on log-transformed data.

CRP, C-reactive protein; HP, *Helicobacter pylori*; AZ, azithromycin; PL, placebo.
